# Safety and immunogenicity of an HIV envelope trimer immunogen that elicits CD4 binding site neutralizing antibody precursors (HVTN 300)

**DOI:** 10.64898/2026.03.31.26349761

**Published:** 2026-04-03

**Authors:** Stephen R. Walsh, William O. Hahn, Wilton B. Williams, Ollivier Hyrien, Pei-Chun Yu, K. Rachael Parks, Robert J. Edwards, Rob Parks, Maggie Barr, Laura L. Polakowski, India Tindale, Megan Jones, Claudio Yurdadon, Randy Burnham, Chen-Hao Yeh, Jack Heptinstall, Kelly E. Seaton, Jessica G. Andriesen, Zachary Sagawa, Maurine D. Miner, Steve De Rosa, M. Juliana McElrath, Lawrence Corey, Georgia D. Tomaras, David C. Montefiori, Barton F Haynes, Kenneth H. Mayer, Lindsey R. Baden, the NIAID HVTN 300 Study Group

**Affiliations:** 1 – Division of Infectious Diseases, Brigham & Women’s Hospital, Boston, MA, United States; 2 - Harvard Medical School, Boston, MA, United States; 3 – Vaccine and Infectious Disease Division, Fred Hutchinson Cancer Center, Seattle, WA, United States; 4 - Duke Human Vaccine Institute, Duke University, Durham, NC, United States; 5 – National Institute of Allergy and Infectious Diseases, Division of AIDS, Bethesda, MD, United States; 6 - Access to Advanced Health Institute, Seattle, WA, United States; 7 - The Fenway Institute and Harvard Medical School, Boston, MA, United States

## Abstract

**Background:**

Induction of HIV envelope (Env)-specific broadly neutralizing antibodies (bnAbs) is considered a key objective for HIV-1 vaccine development. One approach is to vaccinate with HIV Env immunogens that initially target the naïve B cell receptors of a bnAb type and boost with a series of HIV Env variants. We chose a priming immunogen, the CH505 transmitted/founder Env with high affinity for the naïve B cell receptor of the prototype CD4 binding site (bs) bnAb lineage, CH103, as a candidate priming immunogen to induce the initial critical step in CD4bs bnAb development.

**Methods:**

HVTN 300 is a first-in-human, open-label Phase 1 study evaluating the safety and immunogenicity of a CH505 TF chimeric (ch) Trimer adjuvanted with 3M-052-AF (a TLR7/8 agonist) + Alum. The immunogen is a recombinant, stabilized chimeric Env trimer protein with the N-terminal sequence of CH505 TF gp120 Env transplanted into the BG505 SOSIP sequence. Participants received the adjuvanted protein administered in both deltoid muscles at months 0, 2, 4, 8, and 12.

**Results:**

Adults (n=18) aged 18 to 55 were screened at a single site in Boston, USA, and 13 were enrolled. Local and systemic reactogenicity was typically mild to moderate. One participant had severe pain/tenderness, and five participants reported transient severe systemic symptoms at least once. Five participants chose to stop further vaccination due to reactogenicity. No vaccine-related SAEs occurred. Vaccine-specific B-cell response rates reached 100% two weeks post third and fifth vaccinations. Antibody blocking experiments with monoclonal antibodies demonstrated that most participants had antibodies directed to the CD4bs. Four out of 11 participants had serum neutralization signatures for CD4bs bnAb precursors.

**Conclusions:**

No safety concerns were identified. The adjuvanted CH505 TF chTrimer elicited serum antibodies capable of CD4bs-mediated neutralization against strains designed to detect early precursors of the CD4bs B-cell lineages.

**Trial Registration:**

NCT04915768

## INTRODUCTION

A safe and effective vaccine which can prevent HIV-1 acquisition remains elusive and to date, no HIV vaccine has been successful at eliciting broadly neutralizing antibodies (bnAbs) in humans^1,2^. The highly conserved CD4 binding site (CD4bs) is one target for bnAbs that have been isolated from people living with HIV (PLWH) late after acquisition of HIV^3,4^ and fall into two broad classes. One type of CD4bs bnAb binds to the HIV Env and mimics CD4 binding via the antibody complementarity determining region 2 of the immunoglobulin heavy chain (CDRH2)^5^. These antibodies either use a VH1–2*02/04 gene^6^ or use a VH1–46 gene^7^. The second type of CD4bs bnAb uses a wider variety of variable heavy (VH) genes and binds head-on to the CD4bs using CDRH3 loops^8^. The HIV Antibody Mediated Prevention (AMP) trials showed that passive infusion of the CD4 mimicking CD4bs bnAb VRC01 could prevent acquisition of HIV strains highly sensitive to the antibody, thus demonstrating these types of antibodies are desirable to induce with a vaccine^9^.

Work from studies of HIV-bnAb co-evolution have revealed how bnAbs develop sequentially^7,8,10^ and provided a blueprint of a strategy for inducing bnAbs, namely targeting naïve bnAb B-cell receptors (BCRs) followed by sequential immunogens with progressive affinity gradients to guide bnAb maturation from the unmutated common ancestor (UCA)^11–14^. Studies with immunogens designed to target the naïve B cell receptors (BCRs) of CD4 mimicking CD4bs bnAbs have induced VRC01-class B cell lineages with these properties in humanized mice^15–19^ and humans^20–22^. Although no HIV vaccine has yet been successful at eliciting serum antibodies capable of neutralizing diverse circulating HIV strains in humans, Caniels et al.^20^ and Willis et al.^22^ have used germline B-cell receptor-targeting immunogens to induce B cells that may represent the initiation of desirable bnAb B-cell lineages targeting the CD4bs^8,12^. Moreover, B-cell lineages targeting other epitopes have been induced by vaccination, for example a gp41 MPER peptide liposome that neutralized 41% of clade B HIV and 14% of global HIV strains^23,24^. However, to date, no clinical trials have been aimed to elicit the second type of CD4-targeting neutralizing antibodies, the HCDR3-binder type of CD4bs bnAbs.

Here in the HVTN 300 trial (NCT04915768), we tested an immunogen targeting the naïve B-cell receptor of the CDRH3-binder CD4bs bnAbs. The subtype C transmitted founder (TF) strain CH505 was isolated from a PLWH who developed CD4bs-targeting bnAbs of the HCDR3 binder type called CH103^8^. CH103 neutralized 55% of isolates in a panel of 196 globally diverse Env pseudoviruses with a geometric mean IC50 of 4.54 mcg/mL^8^. The CH103-class of CD4bs bnAbs is a leading target for vaccine development because of the lower degree of somatic hypermutation in the CH103 bnAb lineage^8^ and the lower number of improbable mutations required to achieve neutralization breadth^28^ as compared to other CD4 mimicking CD4bs bnAb lineages such as VRC01. CH505 TF stabilized chimeric trimer (CH505 TF chTrimer), a chimeric product containing the N-terminal sequence of CH505 gp120 inserted into a BG505 gp41 SOSIP (i.e., stabilized) trimer sequence, induced CD4bs targeted as well as V1V3 glycan targeted responses when administered with the TLR 7/8 agonist 3M-052 plus alum adjuvants in nonhuman primates (NHP)^29^.

It has been shown in rhesus macaques that adjuvant choice directly impacts tier 2 neutralization, with 3M-052 being superior to the TLR4 agonist, glucopyranosyl lipid adjuvant-stable emulsion (GLA-SE)^29^. Furthermore, in a recent phase 1 clinical trial, it was found that BG505 SOSIP.664 Env trimers adjuvanted with 5 mcg of 3M-052-AF/Alum elicited tier 2 autologous nAbs in serum in three out of five participants^30^. This neutralization was directed to strain-specific glycan holes and no heterologous neutralization was observed, but tier 2 autologous neutralizing had not been observed in any other human trials of Env trimers adjuvanted with alum alone or liposomal MPLA^30,31^.

The primary goals of this first-in-human HIV Vaccine Trials Network (HVTN) trial (HVTN 300) were to determine whether the CH505 TF chimeric (ch) Trimer immunogen combined with a 3M-052-AF adjuvant combined with Alum was: 1) safe and 2) whether it could promote the activation and expansion of precursors of HCDR3-binder type of CD4 binding site bnAbs similar to those known to be associated with bnAbs such as the prototype CH103 CD4bs bnAb induced during natural HIV-1 infection. We found the trimer-adjuvant combination to be safe but frequently reactogenic. Here and in a companion study of induced HVTN 300 B cell repertoires (Chen Hao), we show that the CH505 TF chTrimer was immunogenic and induced HCDR3-binder bnAb precursor B cell lineages in a majority of vaccinees that received 2 or more immunizations.

## MATERIALS AND METHODS

### Study design

HVTN 300 was an open label Phase 1 clinical trial which aimed to enroll at least 12 healthy participants aged 18 through 55 years in Part A (Part B is ongoing and will be reported separately). Each participant received 300 mcg of CH505 TF chTrimer intramuscularly (IM) admixed with 5 mcg of 3M-052-AF and 500 mcg alum in 1 mL total volume (Supp Figure 1). Participants received product on day 0 (month 0), day 56 (month 2), day 112 (month 4), day 224 (month 8), and day 364 (month 12). Half the total volume (i.e., 0.5 mL) was injected into each deltoid by needle and syringe (bilateral injections). Select eligibility criteria included being 18–55 years of age, healthy, living without HIV, at low likelihood of HIV acquisition, and willing to provide informed consent. This study was approved by the study was approved by the Partners (MassGeneral Brigham) Human Research Committee and all participants provided written informed consent.

To assess safety, participants were provided a diary card on which they recorded local and systemic reactogenicity for 7 days post-vaccination. All participants potentially capable of pregnancy had a negative pregnancy test prior to each vaccination. Participants were evaluated for safety and immune responses through blood collection at pre-specified timepoints throughout the study (pre-vaccination and 2 weeks, 10 weeks, 18 weeks, 34 weeks, 54 weeks, and 80 weeks post-vaccination). Leukapheresis was performed pre-vaccination and 34 weeks post-vaccination (after the fourth vaccination). Reactogenicity (solicited adverse events [AEs]) and unsolicited AEs were assessed as per the NIAID Division of AIDS (DAIDS) Table for Grading the Severity of Adult and Pediatric Adverse Events, Corrected Version 2.1, available on the RCC website^32^.

Participants had up to 18 months of scheduled clinic visits and were followed for safety for AEs of special interest (AESI) for 12 months following the last vaccination. Safety of participants was overseen by the HVTN 300 Protocol Safety Review Team and an independent Safety Monitoring Board.

### Study products and administration

The CH505 TF chTrimer is a stabilized chimeric SOSIP Env trimer protein with the N-terminal to the α5 helix sequence of CH505 TF gp120 Env transplanted into the BG505 SOSIP Env trimer sequence^33,34^. Thus, bnAbs targeting the V2 apex, base of the V3 loop, CD4bs, and gp120-gp41 interface can bind to the chimeric Trimer, whereas non-neutralizing antibodies targeting C1 and V2 do not bind. The addition of E64K and A316W mutations eliminates antibody recognition of V3 and the keep the trimer in the stabilized prefusion state^35^. CH505 TF chTrimer protein bound to the CH103 UCA whole mAb at Kd = 61 nM apparent affinity^29^ and the affinity with a Fab 449.3 nM^37^. 3M-052-AF is a lipid-modified aqueous formulation (AF) of the small molecule imidazoquinoline that is a toll-like receptor (TLR) 7/8 agonist. Alum is included in the 3M-052-AF formulation to collocate antigen and adjuvant^29,38^.

### T-cell responses

Flow cytometry was used to examine HIV-1-specific CD4+ and CD8+ T-cell response rates and magnitudes using a previously published validated intracellular cytokine staining (ICS) assay^39^. Previously cryopreserved peripheral blood mononuclear cells (PBMC) were stimulated with synthetic peptide pools and co-stimulated with purified antibodies specific for CD28 and CD49d. As a negative control, cells were stimulated with peptide diluent and co-stimulatory antibodies. As a positive control, cells were stimulated with a polyclonal stimulant, staphylococcal enterotoxin B (SEB). The negative control was plated in two replicates and other stimulations were in singlets. The primary immunogenicity T-cell endpoints were CD4+ and CD8+ T-cell responses, measured by ICS for IFN-γ and/or IL-2 to any Env peptide pool. PBMC cell viability was required to be 66% or greater on the second day after sample thawing.

### B-cell phenotyping

The frequency of CH505-specific B cells was measured by flow cytometry from PBMC specimens obtained at visits 2 (Month 0; baseline), 5 (Month 2.5; 2 weeks post 2nd vaccination), and 7 (Month 6.5; 2 weeks post 3rd vaccination). Live B cells were identified using doublet exclusion, lymphocyte scatter profile, viability dye, and the following lineage markers: negative for CD3, CD56 and CD14, and positive for CD19 and CD20. Memory B cells were further gated on IgD- and IgG+ B cells were additionally gated on IgG+. Detection of CH505-specific B cells was determined based on binding to two fluorescently labeled chCH505 TF 4.1 SOSIP Trimer probes^29^.

### Neutralizing antibody (nAb) assay

nAbs against HIV-1 were measured as a function of reductions in Tat regulated luciferase (Luc) reporter gene expression in TZM-bl cells^40,41^. TZM-bl (also called JC57BL-13) is a HeLa cell clone that was engineered to express CD4 and CCR5^42^ and to contain integrated reporter genes for firefly luciferase and *E. coli* β-galactosidase under control of an HIV-1 long terminal repeat (LTR)^43^. The cells are highly permissive to infection by most strains of HIV, including primary HIV-1 viruses and molecularly cloned Env pseudotyped viruses. Diethylaminoethyl (DEAE)-Dextran was used in the medium during neutralization assays to enhance infectivity. Expression of the reporter genes was induced in trans by viral Tat protein soon after infection. Luciferase activity was quantified by luminescence and directly proportional to the number of infectious virus particles present in the initial inoculum. The assay was performed in 96-well culture plates for high throughput capacity. Use of a clonal cell population provided enhanced precision and uniformity. The assay has been formally optimized and validated for single round infection with either uncloned or molecularly cloned Env pseudotyped viruses produced by transfection in 293T cells^41^.

### Binding antibody multiplex assay (BAMA)

IgG-specific serum antibodies against HIV-1 gp41 and CH505TF SOSIP antigens were measured on a Bio-Plex instrument (Bio-Rad) using a custom HIV-1 Luminex assay^44–46^. Standard positive controls consisted of predefined monoclonal antibodies with known specificity (PGT145, PGT141) and polyclonal sera from PLWH. Standard negative controls of serum from people living without HIV and the CH65 mAb were included at a single dilution. Serum samples were tested across six dilution points at a fivefold serial dilution, with 1:50 dilution as the starting point. The results are presented as an area under the mean fluorescence intensity (MFI) curve (AUC) across the six dilutions, calculated using the trapezoidal rule and with MFI values truncated at zero. A positive response met three criteria around increase in MFI from the baseline response and absolute MFI at the 1:50 dilution. The three criteria were as follows: (1) the MFI minus Blank (MFI*) values were ≥ antigen-specific cutoff at the 1:50 dilution level (based on the 95th percentile of baseline samples as calculated by SAS PROC UNIVARIATE default method, and at least 100 MFI*), (2) the MFI* values were greater than three times the baseline (day 0) MFI* values, and (3) the MFI values were greater than three times the baseline MFI values.

### ELISA plasma binding assay

HIV Env SOSIP trimer binding ELISAs were conducted as follows. 384 well ELISA plates (Costar #3700) were coated with 10μL streptavidin (Thermo Fisher Scientific Inc. #S-888) diluted to 2mcg/mL in 0.1M sodium bicarbonate overnight at 4°C then washed with PBS/0.1% Tween-20 and blocked for 60 minutes at room temperature with assay diluent (PBS/4%(w/v) whey protein/15% Normal Goat Serum/0.5% Tween-20/0.05% Sodium Azide). Plates were incubated for 60 minutes with 10μL of biotinylated Env at 2mcg/mL followed by washing. Plasma samples were titrated in a 12 by 3-fold serial dilution starting at 1:30 then 10μL was added to assay plates for 90 minutes followed by washing. Goat anti-human IgG-HRP secondary antibody (Jackson ImmunoResearch C:109-035-008) diluted 1:15,000 in assay diluent without azide was added at 10μL per well and incubated for 60 minutes. The plates were washed and detected with 20μL SureBlue Reserve (Seracare #5120-0081) for 15 minutes. The reaction was stopped with the addition of 20μL HCL stop solution. Plates were read at 450nm. Using Molecular Devices ‘Softmax-Pro’ software, absorbance values from each 12-point binding curve were used to calculate the Log Area Under the Curve (Log AUC). Baseline Log AUC was subtracted from all time points for each participant sample and plotted as the change in Log AUC (Δ Log AUC).

### Plasma blocking of monoclonal antibody binding to HIV Env

The ability of plasma to block monoclonal antibody binding to HIV Env SOSIP trimer was measured by ELISA in 384 well plates (Costar #3700). Monoclonal antibodies with defined Env binding specificities were labeled with horse radish peroxidase (HRP) using Lightning-Link^®^ kits (Novus Biologicals #701–0003). Plates were coated with 10μL streptavidin (Thermo Fisher Scientific Inc. #S-888) diluted to 2mcg/mL in 0.1M sodium bicarbonate overnight at 4°C then washed with PBS/0.1% Tween-20 and blocked for 60 minutes at room temperature with assay diluent (PBS containing 4%(w/v) whey protein/15% Normal Goat Serum/0.5% Tween-20/0.05% Sodium Azide). Plates were incubated for 60 minutes with 10μL of biotinylated Env followed by washing. Plasma samples diluted to 1:50 were incubated in triplicate wells at 10μL each for 90 minutes followed by washing. The HRP labeled test antibodies were added at 10μL for 60 minutes. sCD4 blocking assays included the additional incubation of sCD4 on the plates for 60 minutes followed by washing prior to the HRP-antibody step. The working concentrations of the biotinylated Envs, sCD4 and HRP labeled antibodies used above were determined in prior optimization assays that allowed for optimal blocking of the unlabeled version of the same antibody. Plates were washed a final time and HRP-antibody binding was detected with the addition of 20μL SureBlue Reserve (Seracare #5120–0081). The reaction was stopped with 20μL HCL stop solution. Plates were read at 450nm. Percent blocking of each sample was calculated using absorbance values as follows: 100-((sample mean absorbance/0% blocking control mean absorbance) × 100).

### Electron microscopy-based polyclonal epitope mapping (EMPEM)

Polyclonal IgG from each subject was isolated from 1 mL aliquots of serum by binding to NAb Protein G Spin Columns (Thermo Scientific, cat. no. 89957), washing with binding buffer, and eluting with IgG elution buffer (Thermo Scientific, cat. no. 21004) into Tris-HCl neutralization buffer (Sigma Aldrich, cat. no. T2694), per manufacturer’s instructions, and buffer-exchanging into PBS with 30-kDa MWCO spin concentrators. IgG was then digested with immobilized papain (Thermo Scientific, cat. no. 20341) per manufacturer’s instructions, using 0.1 mL of resin slurry per 1 mg of IgG and incubating 4–5 hours at 37°C with gentle mixing by inversion. Following digestions, Fab was purified with Protein A Sepharose Fast Flow resin (GE Healthcare, cat. no. 17127902), per manufacturer’s instructions, and buffer-exchanged into PBS with 10-kDa MWCO spin concentrators to yield ~1 mg of polyclonal Fab at ~10 mg/mL concentration. The full amount of Fab was then mixed with 20 μg of CH505 TF SOSIP trimer and incubated overnight at 4°C. Following incubation, each sample was fractionated by size exclusion chromatography over a Superose 6 Increase 10/300 column, and fractions from the peak presumed to be the Env-Fab complex were pooled and concentrated in a 100-kDa MWCO spin concentrator. Concentrated samples were diluted to 0.1–0.3 mg/mL in buffer containing 20 mM HEPES, pH 7.4, 150 mM Na_2_SO_4_, 2.5 mM NaCl, 2.8 mM NaN_3_, and 8 mM glutaraldehyde. After 5 min incubation, excess glutaraldehyde was quenched by adding sufficient 1 M Tris stock, pH 7.4, to give 80 mM final Tris concentration and incubated for 5 min. Quenched samples were applied to a glow-discharged carbon-coated EM grid for 10–12 second, then blotted, and stained with 2 g/dL uranyl formate for 1 min, blotted and air-dried. Grids were examined on a Philips EM420 electron microscope operating at 120 kV and nominal magnification of 49,000×, and ~100–400 images were collected on a 76 Mpix CCD camera at 2.4 Å/pixel. Images were analyzed by 2D class averages, 3D classification, and 3D refinements using standard protocols with Relion 3.0^47^.

### Statistical Methods:

Safety outcomes were evaluated among all enrolled participants. Immunogenicity outcomes after a given dose were evaluated among participants who received all doses up to that time point.

### Neutralization analysis:

Criteria for positive nAb responses were titer > 10 and for Env ELISA responses were titer ≥ 100; Ab responses below the lower limit of quantitation (LLOQ) of the assay were imputed to the numeric value of the LLOQ. Differential neutralization of a precursor detection virus and its corresponding epitope knock-out virus was calculated by dividing the ID50 or ID80 titer against the precursor detection virus by the respective ID50 or ID80 titer against the epitope knock-out virus. A difference of 3-fold or greater was considered positive for the presence of a neutralization signature of bnAb precursors.

### Analysis of B- and T-cell positive responses:

To determine response positivity for a peptide pool within a T-cell subset at a given timepoint for each participant, a two-by-two contingency table was constructed to compare the frequency of cytokine-producing T cells in the HIV-1 peptide-stimulated sample with that in the corresponding negative control. A one-sided Fisher’s exact test was applied to the table to test the null hypothesis that the frequency of cytokine-producing T cells in the stimulated sample was equal to that in the unstimulated sample against the alternative hypothesis that it was greater. Since multiple tests (for each peptide pool) were conducted, a multiplicity adjustment was performed using the Bonferroni-Holm adjustment procedure to correct the individual peptide pool p-values. Because the total cell counts for the T-cell subset were generally large (e.g., as high as 100,000 cells), the Fisher’s exact test was expected to have high power to reject the null hypothesis, even for small differences. Therefore, the p-value significant threshold was chosen stringently (≤ 0.00001), and a response was declared positive if the resulting p-value was ≤ 0.00001.

Similar to the T-cell data above, a two-by-two table contingency table was constructed using post vaccination and baseline data. The four entries in each table were the numbers of Env-specific B cells and total B cells after vaccination and at baseline. A one-sided Fisher’s exact test was applied to the table to test the null hypothesis that the frequency of Env-specific B cells post vaccination was equal to that at baseline against the alternative hypothesis that it was greater. A response was declared positive if the resulting p-value was ≤ 0.00001.

For both T and B cells, the rate of positive responses was estimated for each timepoint as the empirical frequency of participants with a response declared positive.

## RESULTS

### Study population

Adults (n=18) aged 18 to 55 were screened at a single site in Boston (USA) and 13 participants were enrolled into HVTN 300 (Supplemental Figure 1). In total, 76.9% (10/13) of participants were male (Supplemental Table 1). Two of 13 (15.4%) were Hispanic/Latine, 11/13 (84.6%) were White, and the median age was 34 years (range 21–46). Seven participants took PrEP (either TAF/FTC [3] or TDF/FTC [4]) for at least some part of the study follow-up. There were no pregnancies nor HIV acquisition events during the study. Safety outcomes were evaluated among all enrolled participants. Immunogenicity outcomes after a given dose were evaluated among participants who received all doses up to that time point.

### Safety and tolerability

Thirteen participants were enrolled. One participant decided to forgo further study product administrations after the first administration due to a panic attack immediately following the first vaccination. One participant was lost to follow-up after the first vaccination. Eleven participants received the second vaccination, 10 participants received the third vaccination, and 9 participants received the third and fourth vaccinations, and 7 received all five scheduled vaccinations. In total, four participants discontinued due to transient solicited adverse events, one discontinued due to a panic attack, and one was lost to follow-up. See Supplemental Figure 2 for details of vaccine completion.

**Figure 1.**
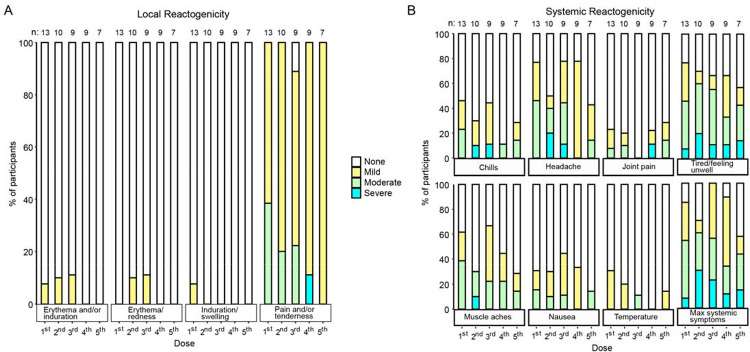
Local (A) and systemic (B) reactogenicity in HVTN 300. The maximum local (left) and systemic (right) solicited adverse events reported by participants within seven days after each. Local events include injection site pain and/or tenderness, erythema, and induration. Systemic events include: malaise and/or fatigue, myalgia, headache, nausea, chills, arthralgia, fever. All grading was per the DAIDS Table for Adverse Events Version 2.1^32^ and was reconciled by a clinician.

Local reactogenicity occurred in almost all participants (Figure 1A) but was mostly mild to moderate. One participant experienced severe pain/tenderness at both the right and left injection sites 3 days following the fourth vaccination, though it lasted only one day.

Nearly all participants reported some systemic reactogenicity during the course of the trial, mostly mild-to moderate (Figure 1B). For example, 9 of 13 participants experienced systemic reactogenicity after the first dose. There were four participants who reported systemic reactogenicity as severe throughout the trial. One of these participants experienced subsequent vaccinations without any Grade 3 reactogenicity, one participant experienced an additional repeat Grade 3 systemic reactogenicity, and two participants declined to receive additional vaccinations after the first Grade 3 systemic reactogenicity event. Most reactogenicity events (both systemic and local) resolved within 2 days and all Grade 3 within 7 days.

Only one participant reported an unsolicited adverse event that was deemed related to study product (transient skin sensitivity for up to 3 days after vaccination). Other unsolicited AEs not related to study product are listed in Supplemental Table 2. There were no related serious AEs (a classification distinct from severe), adverse events of special interest (defined as prespecified immune mediated diseases), or deaths.

### T-cell responses

**Figure 2.**
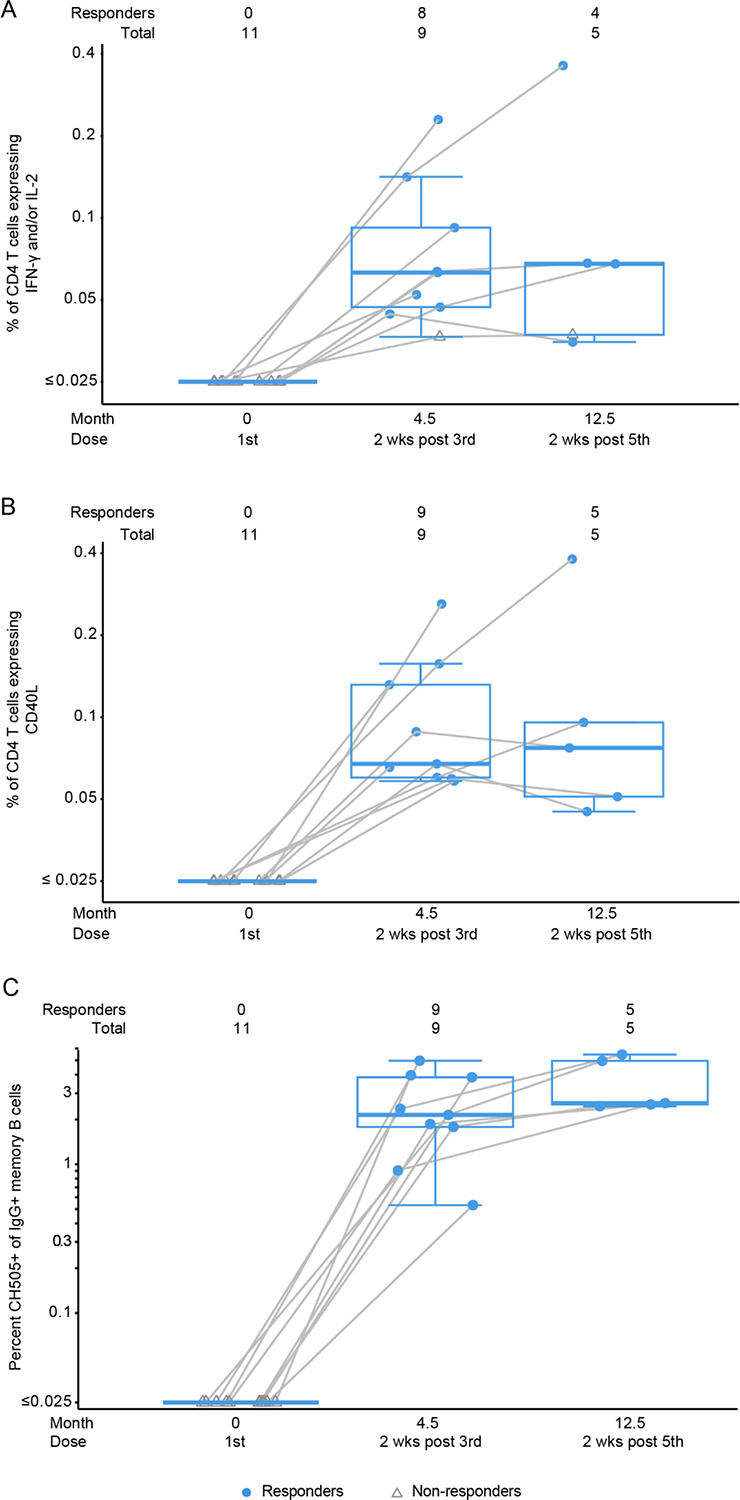
Vaccine-specific CD4+ T cells (A, B) and memory B cells (C) elicited by vaccination do not increase between third and fifth vaccination. Vaccine-specific CD4+ T cells expressing IFN- and/or IL-2 (A) or CD40L (B). (C) Vaccine-specific memory B cells. Boxplots were constructed using data from all participants, with positive responders indicated in color and non-responders in gray. Data points for each participant are connected by a gray line. The mid-line of the box denotes the median and the ends of the box denote the 25th and 75th percentiles. The whiskers that extend from the top and bottom of the box extend to the most extreme data points that are no more than 1.5 times the interquartile range (i.e., height of the box) or if no value meets this criterion, to the data extremes.

The response rates of CD4+ T cells expressing IL-2 and/or IFN-γ to CH505 TF gp140 at month 4.5 (two weeks post 3^rd^ vaccination) and month 12.5 (two weeks post 5^th^ vaccination) were above 80% at both timepoints and reached median magnitudes of around 0.05%, with no increase between the 3^rd^ and 5^th^ dose (Figure 2A). CD8+ T-cell response rates and magnitudes were negligible, with positive response rates (e.g., detectable production of IFN-γ and/or IL-2) in 2 of 9 and 1 of 5 of participants at month 4.5 and 12.5, respectively (Supplemental Figure 3A).

### B-cell responses

All 9 participants developed memory B cells capable of binding the CH505 TF Env trimer after the third vaccination (Figure 2B). As a percentage of IgG+ memory B cells, the median response magnitude was 1.9% after three vaccinations and did not increase substantially after the fifth dose in the 7 individuals who received 5 immunizations. (Figure 2B). No vaccine-specific IgM memory B cells were identified above background at these timepoints and only one participant had IgA memory B cells detectable above background (Supplemental Figure 3C).

### Binding antibody responses and specificities

**Figure 3.**
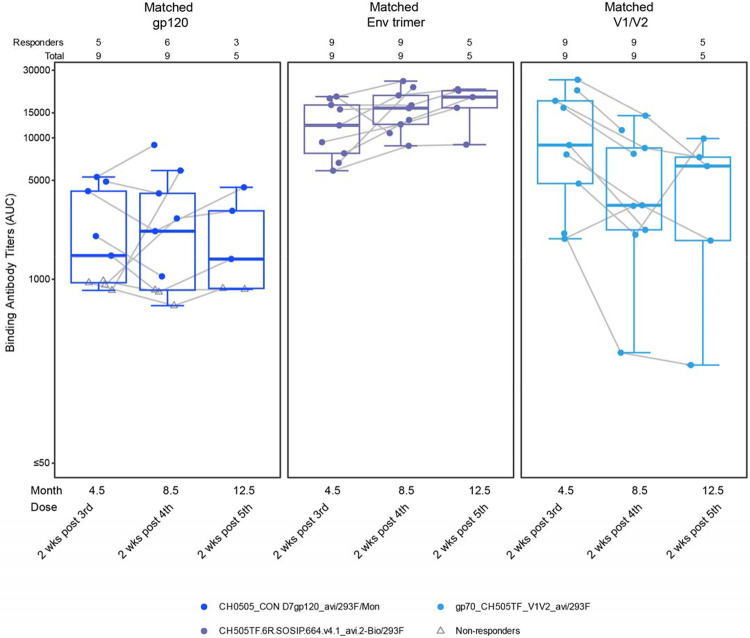
Vaccine-specific SOSIP trimer binding antibodies elicited by vaccination. Specific responses were measured by BAMA in serum preimmunization and 2 wk following the indicated dose of vaccine to the following analytes: a gp120 matched to the vaccine (CH505 TF), a trimer matched to the vaccine (CH505TF SOSIP), and V1/V2 (CH505).

Binding antibodies were assessed using BAMA against a panel of analytes. These included binding to the vaccine matched trimer (“CH505TF.6R.SOSIP.664v4.1”), the gp120 portion of the vaccine (“CH505_CON D7 gp120”), and a V1/V2 gp70 protein that measures non-neutralizing V2 antibodies implicated as protective in the RV144 efficacy trial^44^. All vaccinees exhibited serum including antibodies capable of binding the vaccine-matched trimer, a vaccine-matched V1/V2 scaffold, and most participants demonstrated antibodies capable of binding the gp120 portion of the vaccine-matched Env (Figure 3). Interestingly, binding antibody responses appeared to peak after the third dose, with no evidence of boosting against the matched trimer or gp120 subunit with doses 4 and 5. Antibodies against the V1/V2 scaffold waned after the 5^th^ vaccination..

To test whether serum antibodies were directed against the CD4bs, two knock-out Env gp120 mutations were used in BAMA: either D368 (an epitope important for CH103-and-VRC01 lineage binding) or D371 (an epitope important for most CD4bs antibodies) using the validated BAMA. Only one participant had evidence of differential binding to the D371 epitope at Month 4 (Supplemental Figure 4B). This participant also had evidence of differential neutralization (see below). No participant had differential binding of D368.

### Neutralizing antibody responses to tier 1 and 2 viruses

**Figure 4.**
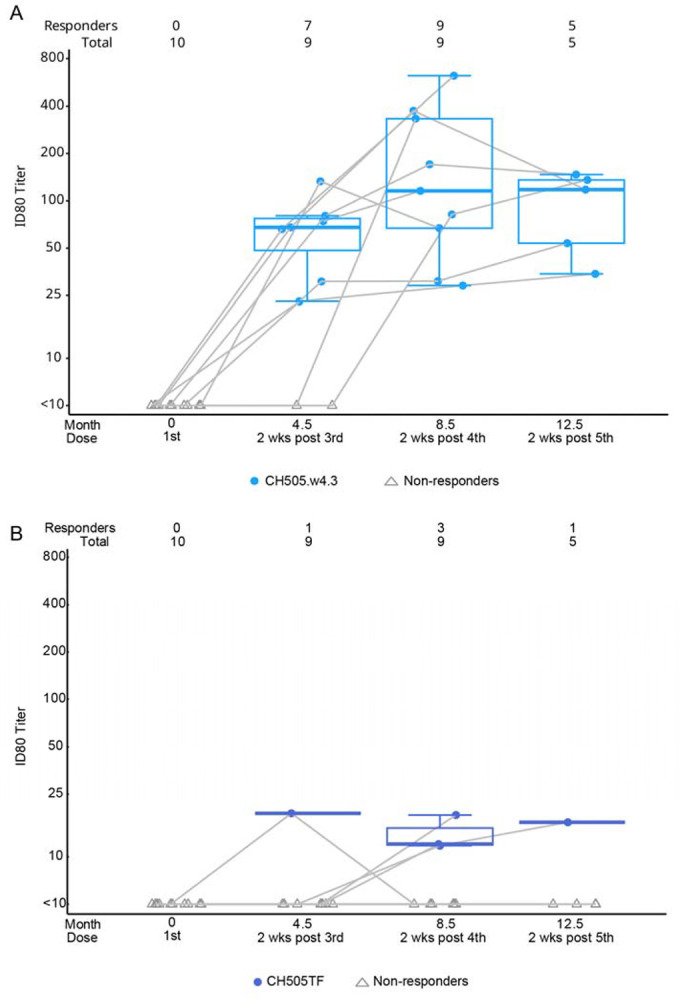
Tier 1 & 2 neutralizing antibody responses. ID80 neutralizing antibody titers against the autologous Tier 1 (CH505.w4.3) and autologous tier 2 HIV-1 (CH505TF) using the TZM-bl neutralization assay prior to the first vaccination and after the indicated vaccine dose.

Using the TZM-bl pseudovirus assay, we measured nAb responses to autologous tier 1 and tier 2 viruses. Most participants had detectable nAb ID80 titer response rates to autologous tier 1A isolates at all timepoints tested post-vaccination with no evidence of nAb titer boosting between the third and fifth vaccination (Figure 4A). Autologous tier 2 nAb ID80 responses (matched to the vaccine trimer) were detected in 3/9 study participants after the fourth vaccination (Figure 4B). The autologous tier 2 CH505 TF nAb responses were first detected at month 4.5 in 1 participant, and month 8.5 in 3 participants (including the one participant with detectable activity at month 4.5). Responses declined to <10 at month 12.5 in 2 out of these 3 participants. Samples testing positive against the autologous virus were assayed against a global panel of 9 heterologous tier 2 viruses. All results were negative except for 1 serum sample that exhibited weak activity against 2 heterologous viruses, TRO.11 Y and 25710–2.43 (ID50=10 and 13, respectively) at month 12.5 only. This latter sample also had a low neutralization titer against the tier 2 autologous CH505 TF virus (ID50=14).

### Electron Microscopy-based Polyclonal Epitope Mapping (EMPEM)

**Figure 5.**
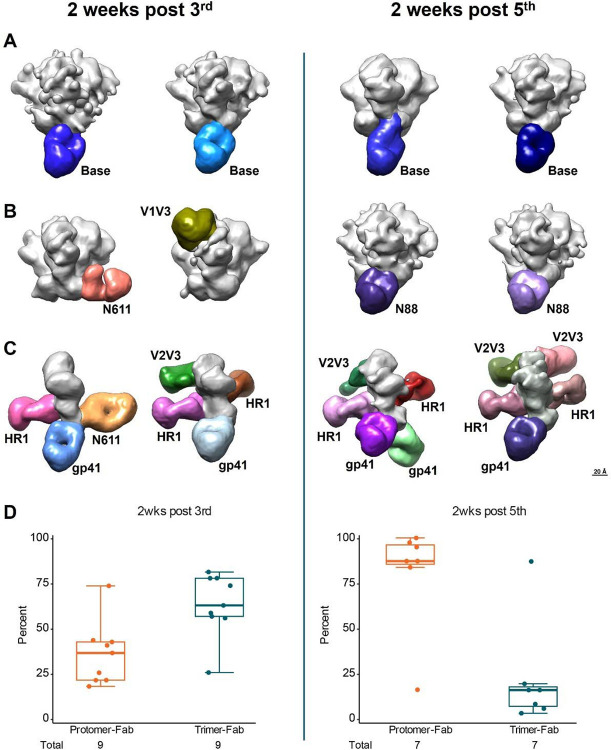
Polyclonal Fab fragments mostly bind to base or internal protomer epitopes, while the percentage of gp120-gp41 protomer dissociation increases with additional vaccinations. (A) Examples of polyclonal Fabs (pFabs) targeting the trimer base, with the Env trimer in gray and the pFabs in shades of blue from two weeks post third vaccination (left) and two weeks post fifth vaccination (right). (B) Example pFabs targeting the N611 glycan region (salmon), the V1V3 region (olive), and the N88 glycan region (shades of purple). Note, the structure showing a pFab targeting the V1V3 region comes from subject 300–276850, who only received two vaccinations; thus, this structure is from 10 weeks post second vaccination. (C) Examples of pFab-bound protomers. Env protomers (gray) are shown in the same orientation as the protomer closest to the viewer in the trimer structures in A-B. At least four internal epitopes can be identified, two in the beta-hairpin regions of the V3 and V3 loops (labeled V2V3), and two in the HR1 region of the gp41 (labeled HR1). In the context of an intact trimer, these epitopes would be inaccessible to antibodies. One pFab (orange) is tentatively identified as targeting the N611 glycan region. The remaining protomer-bound pFabs appear to be targeting either the base epitope or the N88 glycan region, however, because there are no published high-resolution structures of isolated gp41-gp120 SOSIP protomers to compare these NSEM maps to, these pFabs are only identified and labeled as gp41-directed. Scale bar at lower right conveys for A-C. (D) Percentages of Fab-bound trimer particles and Fab-bound protomer particles from EMPEM data.

To analyze the specificity and time-course of the serum IgG response we performed electron microscopy based polyclonal epitope mapping (EMPEM) after the third and fifth vaccinations. At both time points, we observed qualitatively that most trimer-Fab binding was directed to the trimer base. Representative images are shown in Figure 5A. In addition to the trimer base-binding, we also observed a small minority of Fab-binding directed to gp41 in the region of the N611 glycan and to gp120 in the V1V3 region of the trimer apex after the third vaccination, and to gp41 in the region of the N88 glycan after the fifth vaccination (Figure 5B). At both time points, we observed abundant Fab-binding to individual Env gp120-gp41 protomers, suggesting possible antibody-driven dissociation of the trimers^48^. In a few cases we were able to obtain interpretable protomer-Fab structures, and these indicated at least four additional epitopes that would be internal and hidden in an intact trimer (Figure 5C). The newly exposed epitopes included two epitopes in the beta-hairpin regions of the V3 and V3 loops (labeled V2V3), and two epitopes in the HR1 region of the gp41 (labeled HR1). Quantifying the relative number of Fab-bound trimer particles versus Fab-bound protomer particles from the EMPEM data, we noted an increase in Fab-protomer binding in most subjects from the third to the fifth vaccination (Figure 5D). The EMPEM data suggests that after the fifth vaccination the most prevalent serum IgG response was directed to internal protomer epitopes or non-neutralizing trimer base epitopes. We did not observe serum IgG binding to the CD4bs by EMPEM at either time point.

### Screening for Polyclonal Antibodies Against Epitopes of Interest

To further characterize serum Env binding activity, we performed a series of blocking experiments testing whether the polyclonal serum could block the binding of monoclonal antibodies with known binding properties. We found that HVTN 300 serum from 7 of 7 participants had antibody capable of blocking soluble (s) CD4 binding to Env and the mature CH103-lineage bnAb, CH106, by an average of 59% and 45% respectively. Six of seven participants were able to block the CH103-lineage UCA an average of 42%

**Figure 6.**
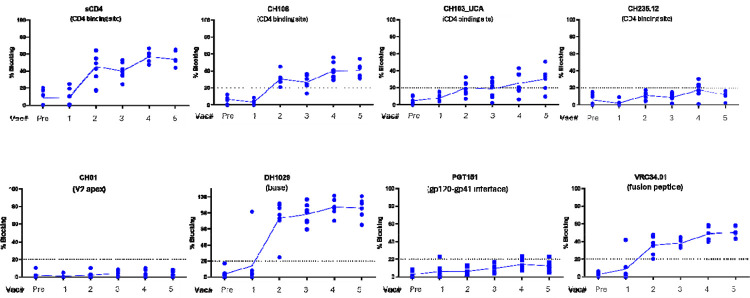
Evidence of CD4 binding site antibodies by competition assay. Env trimers were pre-incubated with serum at dilution of 1:50 drawn two weeks following the indicated vaccination or prebleed timepoints. After washing, the indicated monoclonal antibody labelled with a detector reagent was applied at a half-maximal concentration (see Methods).

Next, we determined that a trimer base-binding antibody (DH1029) had reduced binding when the target trimer was pre-incubated with 7 of 7 participants’ serum (Figure 6). Some blocking was observed by HVTN 300 serum for the fusion peptide bnAb, VRC34, binding to Env in 7 of 7 participants, but none was observed (0 of 7 participants) with PGT151, an antibody that binds to the gp120/gp41 interface of intact trimers^49^. No blocking by any HVTN 300 serum samples of an apex-directed bnAb (CH01) was noted (Figure 6).

As we had observed blocking of the CD4bs but minimal evidence of CD4bs Abs using EMPEM, we investigated the binding properties of polyclonal serum using additional exploratory ELISA assays comparing the binding of candidate Env with specific KO in the CD4bs. These included the following: 1) G367R^50^, a mutation used to identify 12A21-like and VRC01-like antibodies; 2) N280D, a mutation that reduces the binding of early CH235-like antibodies^51^; and 3) the combination S364K/T455E/G459E mutations (“STG”)—a series of mutations deep within the CD4bs that abrogates/reduces binding of many CD4bs bnAbs such as VRC01 and CH103^52^. In the 7 participants who received all 5 vaccine doses, there was evidence of differential reduced binding to each of these knock-out Envs after the third, fourth, and fifth vaccinations indicating the presence of a polyclonal response to the CD4bs. In one individual, the degree of reduced binding to knock-out Envs increased with the 5^th^ immunization (Supplemental Figure 5).

### Serum neutralization of HIV variants susceptible to CD4bs antibodies

**Figure 7.**
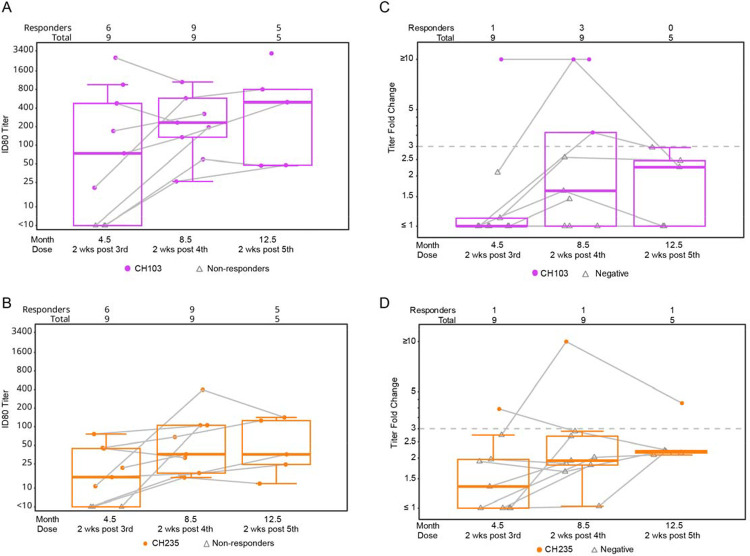
CD4bs-precursor neutralizing antibodies detected in subset of participants and frequency does not increase with additional vaccinations ID80 neutralizing antibody titers against two pseudoviruses designed to be susceptible to CD4bs-directed neutralization using the TZM-bl neutralization assay prior to the first vaccination and after the indicated vaccine dose. The top (A, C) represents CH505TF.gly4/293S (grown in GnT1- cells) and a corresponding strain with an additional mutation (S365P) intended to suggest CH103-like neutralization. The bottom (B, D) represents CH505TF.N279.G458K.D293S (grown in Gn1- cells) and a corresponding strain with a specific knockout in the epitope of interest (N280D) intended to show CH235-like neutralization. A,B show the titers for the indicator virus and C,D show the ratio of titers, indicator vs. KO.

Since we had observed evidence of binding to the CD4bs via blocking and binding experiments, clear differential binding with D371 in one participant and no participants with differential binding to D368, but more clear reductions using other epitopes (G367R, N280D, “STG”) we wanted to determine whether the polyclonal CD4bs antibodies were functionally capable of neutralization. We therefore tested for a reduction in serum activity with the disruption of these epitopes using a two-part assay. The first part of the assay determined neutralization against a pseudovirus engineered to be susceptible to a specific class of CD4bs antibody (either CH505gly4 for CH103-like antibodies or CH505TF.G458Y.N279K for CH235-like antibodies). Since neutralization can be directed at areas outside the CD4bs (e.g., strain specific-glycan holes), a confirmatory assay was used that has a targeted disruption in an epitope known to be important for either HCDR3-binder CD4bs CH103-like neutralization (S365) or CD4 mimicking VH1–46, CH235-like neutralization (N280D). We found two HVTN 300 participants had a strong neutralization signature for CH103 precursors. A third participant had a strong neutralization signature for CH235 CD4 mimicking CD4bs bnAb. A fourth participant had a modest neutralization signature for precursors of both lineages (Figure 7). Coupled with the blocking data of CD4bs bnAb and VRC34 bnAb serum blocking, these serum mapping data suggested that the CH505 TF chTrimer Env induced antibodies targeting the CD4bs and the fusion peptide binding site that were capable of neutralizing HIV pseudovirus strains designed to identify CD4bs bnAb precursors.

## DISCUSSION

This first-in-human administration of CH505 TF chTrimer adjuvanted with 3M-052 AF/alum demonstrated that the combination was immunogenic and safe although local reactogenicity was not well tolerated as 5 participants stopped injections before the trial was complete. We demonstrated that vaccine specific blood memory B cells were elicited in all participants, and 4 participants had plasma antibodies that neutralized autologous tier 2 CH505 TF virus. A subset of participants (4/9) had a neutralization signature in polyclonal serum that was suggestive of on-target neutralization to the HCDR3-binder type, one of two bnAb lineages induced in the CH505 PLWH. Notably, neutralization activity directed against potential bnAb sites was not detected at the polyclonal level in previous trials using other relatively unmodified, native like trimers such as the BG505 SOSIP.664^30^, the BG505 SOSIP trimer with stabilizing modifications^53^, or other Env trimers based upon consensus sequences^31^. Similar to studies of other soluble Env trimers, most serum antibody activity from HVTN 300 participants that was directed against the CH505 TF chTrimer targeted the base of the trimer (as assessed by EMPEM and serum Ig blocking assays). As repeated administration of the CH505 TF chTrimer did not elicit any heterologous tier 2 neutralizing activity in the serum (and only very modest autologous neutralization), the HVTN 300 regimen is unlikely to be advanced as a standalone vaccine. We determined that many of the challenges associated with vaccination with HIV Env trimers could not be overcome with additional dose administrations (up to 5 in this study). It is plausible that additional administrations could be a mechanism for allowing subdominant epitopes to emerge due to vaccination-induced, antibody-mediated blocking of immunodominant, off-target epitopes. Indeed, in NHP models, serum neutralization continually rose over time with each administration beyond the third vaccination with the 3M-052 AF adjuvant^29^. Large “jumps” in neutralization have also been observed with repeated Env immunizations in guinea pigs^54,55^ and tier 2 serum neutralization emerged following the third vaccination of another trimer^30^. Despite these data in preclinical models, additional homologous dosing of these trimers resulted in no increase in the breadth of heterologous or induction of autologous neutralization, and no increase in the number of participants with “on track” responses at the serum level. None of the immune readouts were substantially increased between the third and fifth dose with no difference in the quantity of vaccine specific B cells or CD4+ T cells. The EMPEM data point to an explanation for this lack of boosting of on track responses of the exposure of epitopes on the trimer interior due to vaccine-elicited antibodies that disrupt the trimer^48^. After the fifth vaccination, the majority of observed particles by NSEM were protomers, suggesting that strategies based upon repeated Env trimer administrations should make attempts to reduce the base-directed response and to reduce the number of boosts with the same immunogen. We did observe that some of the antibodies generated against Env epitopes exposed by dissociation of gp120-gp41 bound to the heptad region 1 of gp41. Further investigations will be required to see if antibodies induced by these internal Env epitopes resemble antibodies capable of neutralizing via binding this region that have been isolated in the setting of chronic HIV^56^.

Thus, additional modifications to trimeric Env will be required to efficiently prime bnAb B cells. There may be a point of diminishing returns with homologous Env vaccination, especially trimers lacking additional stabilizing properties and sequential immunization strategies are likely to be required to induce bnAbs in serum. Moreover, the adjuvant formulation will need to be optimized as well.

The additional administration of the CH505 TF chTrimer formulated with 3M-052 AF/alum led more participants to discontinue (5/11) as compared to a three dose regimen using an Env trimer with the same 5 mcg dose of 3M-052 AF/alum^30^. Thus, less reactogenic adjuvants or lower doses of 3M-052 are being explored in additional studies.

That we did not see evidence of CH235-like VH1–46-class antibodies is consistent with data that the CH505 TF does not bind to the CH235 UCA^57^. In this regard, we have shown that a modified CH505 Env trimer engineered to bind well to the CH235 UCA^57^ does induce CH235 precursors and uses the VH1–105 gene that is an ortholog of human VH1–46^51^.

One challenge of using native-like Env trimers to elicit B cells with the potential to produce bnAbs is that conceivably there are many potential solutions the immune system could develop in order to bind to a particular epitope of interest. This is in contrast to more targeted germline approaches where a specific B-cell class is targeted. Many vaccine clinical trials designed to elicit bnAbs use immunogens not expected to induce bnAbs, but instead prime B-cell responses that may be refined to ultimately induce bnAbs^20,21,27^. Recently, a BG505-trimer with extensive modifications intended to target VRC01-class B cells has been demonstrated to elicit a bnAb precursor response in most participants given the same dose of the immunogen utilized in the present trial^20^; 17/18 had a >2.5 fold decrease in serum activity with a knock-out of the CD4bs epitope suggestive of CD4bs-mediated neutralizing activity (in contrast to the 4/11 with a serum signature suggestive of CD4bs-mediated neutralization in our cohort). Because EMPEM is unlikely to reveal binding of low-abundance Fabs, this suggests potential antibodies responsible for the CD4bs neutralization signature are low abundance in serum IgG. Notably, the GT1.1 immunogen elicits a VH1–2 predominant response, whereas the CH103 lineage utilizes the VH4–59 family of genes to bind via CDR3, suggesting potential complementarity in the approaches.

We have shown in a previous HVTN clinical trial, HVTN 133, that design of an immunogen targeted to one unmutated ancestor induced a polyclonal bnAb precursor response to the gp41 membrane proximal external region (MPER) bnAb epitope in gp41^24^. Here, we immunized with the CH505 TF Env chosen for binding to the CH103 unmutated common ancestor naïve BCR, and induced CD4bs serum signature responses in three individuals, suggesting that the responses to target one specific bnAb B-cell lineage are generalizable^24^. Similar to other clinical trials of HIV vaccines^24,53,58^, we anticipate that there will likely be more participants with the desired vaccine-induced B-cell response when the B cells of our participants are tested using clonal B-cell repertoire interrogation methods^24^. For the HVTN 300 participants, the analysis of B cell repertoires of 11 participants in the HVTN 300 are reported in another paper and indeed demonstrated CH103-like HCDR3 binder CD4bs bnAb B cell lineages in 7/10 vaccinees that received more than 2 immunizations, and isolated a clone of fusion peptide proximal region-targeted antibodies that were predicted from the serum responses reported here (Chen Hao, bioRxiV).

In summary, the HVTN 300 clinical trial established that repeated immunizations with a single trimeric Env with a potent adjuvant likely induced necessary B-cell lineages to ultimately enable B cells to mature to broad neutralization, advancing understanding of how to develop a preventative HIV vaccine. It is notable that low-level autologous neutralization was observed with the soluble protein adjuvanted with 3M-052AF/alum, similar to previous results with the BG505 SOSIP trimer in HVTN 137. Overall, the results from HVTN 300 contributed important technical and operational improvements within the context of the early phase discovery medicine program.

## Supplementary Material

Supplement 1
