# Supplementary material for: Safety and immunogenicity of an HIV envelope trimer immunogen that elicits CD4 binding site neutralizing antibody precursors (HVTN 300)
Source: medRxiv. 2026 Apr 3:2026.03.31.26349761. Preprint. [Version 1] doi: 10.64898/2026.03.31.26349761 (PMC13060434; doi:10.64898/2026.03.31.26349761)

SUPPLEMENTAL MATERIAL

Supplemental Table 1. Participant demographics.

Characteristic	N	Female<br>N = 3[1]	Male<br>N = 10[1]	Overall<br>N = 13[1]
**Age**	13	36.0 (30.0 - 39.0)	34.0 (21.0 - 46.0)	34.0 (21.0 - 46.0)
**Race**	13			
American Indian/Alaska Native		0 (0%)	1 (10%)	1 (7.7%)
Black or African American		0 (0%)	1 (10%)	1 (7.7%)
White		3 (100%)	8 (80%)	11 (85%)
**Ethnicity**	13			
Hispanic or Latino		0 (0%)	2 (20%)	2 (15%)
Not Hispanic or Latino		3 (100%)	8 (80%)	11 (85%)

[1]Median (Min - Max); n (%)

Supplemental Table 2. List of AEs.

Participant Pub ID	AE Number	Adverse Event Description	Visit AE First Reported	Severity	Related to Study Treatment	Action Taken with Study Product Administration	Treatment for AE?	Con Med for AE?	MAAE?	Event Outcome	MedDRA PT	Comments
469	1	Hypertension	3	Grade 2 (Moderate)	Not related	No Change	No		No	Recovered/resolved	Hypertension	Grade 1 hypertension at enrollment. Grade 2 increased diastolic blood pressure at visit 3. Referred to PCP. 149/105.
469	2	Conjunctivitis	4	Grade 2 (Moderate)	Not related	No Change	Yes	Yes	Yes	Recovered/resolved	Conjunctivitis	bilateral conjunctivitis. treated with erythromycin ophthalmic ointment with solution. Partner has similar symptoms.
469	3	Hemorrhoid	4	Grade 1 (Mild)	Not related	No Change	Yes	Yes	No	Recovered/resolved	Haemorrhoids	small hemorrhoid- treating with topical ointment. encouraged to increase fiber
469	4	ocular migraine	Interim Visit	Grade 3 (Severe)	Not related	No Change	Yes		Yes	Recovered/resolved	Ophthalmic migraine	While eating soup, participant developed "pixilated blurry vision in the bottom right corner" of his right eye. He presented to ED. Visual changes resolves after 30min. Had mild headache. Normal CT scan, normal eye pressure and eye exam. Diagnosed with ocular migraine, although could not make out TIA. Upon arriving home later, headache became severe. Mild nausea and moderate malaise related to Headache. Seen by ophthalmologist who agrees with diagnosis of ocular migraine. No meds given. Patient states history of migraines over a decade ago, never with

												visual component. Plans to follow up with PCP. Spoke with participant and they confirmed migraine resolved.
67	1	Elevated Creatinine	9	Grade 1 (Mild)	Not related	No Change	No		No	Recovered/resolved	Blood creatinine increased	
67	2	Elevated Blood Pressure	9	Grade 1 (Mild)	Not related	No Change	No		No	Recovered/resolved	Blood pressure increased	PT has BP of 136/91 during visit 9 which is the only reading recorded.
67	3	Monkeypox	Interim Visit	Grade 3 (Severe)	Not related	Not applicable	Yes	Yes	Yes	Recovered/resolved	Monkeypox	impact on daily life: increased fatigue, slept as much as 12 hours a day, moderate fatigue. Quarantined because of caution no related to symptoms. Decreased self care as a result of feeling frustrated not being physically incapable. Pain intensity mild.
86	1	Covid-19	Interim Visit	Grade 2 (Moderate)	Not related	No Change	Yes	Yes	Yes	Recovered/resolved	COVID-19	Tested positive. Onset of conjunctivitis, sore throat and cough, loss of taste and smell. Received monoclonal antibody infusion. Subject reports all symptoms have resolved completely.
86	2	Asymptomatic Pharyngeal Gonorrhea	4	Grade 2 (Moderate)	Not related	No Change	Yes	Yes	Yes	Recovered/resolved	Oropharyngeal gonococcal infection	patient tested after exposure. pharyngeal culture positive. asymptomatic. medically attended.
86	3	Asymptomatic Rectal Chlamydia	6	Grade 2 (Moderate)	Not related	No Change	Yes	Yes	Yes	Recovered/resolved	Anal chlamydia infection	
86	4	Asymptomatic Rectal Chlamydia	8	Grade 2 (Moderate)	Not related	No Change	Yes	Yes	Yes	Recovered/resolved	Anal chlamydia infection	Asymptomatic Rectal Chlamydia found on routine screening. Treated with Doxycycline for seven days.
332	1	Bites- insect multiple Right arm	Interim Visit	Grade 1 (Mild)	Not related	No Change	No		No	Recovered/resolved	Arthropod bite	subject sent images assessed to be insect

												bites. not treated. Resolved.
192	1	Menorrhagia	3	Grade 1 (Mild)	Not related	No Change	No		No	Recovered/resolved	Heavy menstrual bleeding	Reports increased from baseline heavy menstrual bleeding started day after enrollment and lasing 4 days. No interference with activities of daily living, no medications taken. Not medically attended.
192	2	URI	5	Grade 1 (Mild)	Not related	No Change	No		No	Recovered/resolved	Upper respiratory tract infection	URI with concurrent chest congestion. mils. not treated
192	3	Right Eye Keratitis	6	Grade 2 (Moderate)	Not related	No Change	Yes	Yes	Yes	Recovered/resolved	Keratitis	mild right eye redness and mild photophobia for approximately 1 month that worsened which prompted participant to be seen in ER. Wears contact lenses daily for 12+ hours. exam reveals possible keratitis. prescribed ABX eye drops. Subject reports that she took moxifloxicin for one day, traded contacts for glasses, and it cleared up 4 days later.
192	5	Elevated Blood Pressure	8	Grade 1 (Mild)	Not related	No Change	No		No	Recovered/resolved	Blood pressure increased	Asymptomatic elevated diastolic blood pressure at baseline and post-vaccination. Will recheck at next visit. Diastolic blood pressure was recorded as: 90, 92 mm/hg.
192	6	Ovarian Cyst	Interim Visit	Grade 2 (Moderate)	Not related	Not applicable	No		Yes	Recovering/resolving	Ovarian cyst	Participant went to the ER for abdominal pain. A 6cm ovarian cyst was noted. Participant noticed a 15lb weight gain with no life style changes. Participant is looking for a surgical

												gynecologist to explore treatment options.
123	1	Low absolute neutrophil count	5	Grade 2 (Moderate)	Not related	No Change	No		No	Recovered/resolved	Neutrophil count decreased	on visit 5, absolute neutrophil count at 760. Asked to return for a redraw. came in for repeat CBC, which was normal. Participant denies any recent illnesses.
123	2	Right axillary lymphadenopathy	7	Grade 1 (Mild)	Not related	No Change	No		No	Recovered/resolved	Lymphadenopathy	The subject reported at this same visit 07 the receipt of 2 vaccines, annual Flu vaccine as well as the Pfizer COVID-19 boost vaccine. Both of these vaccines were administered which is most likely related to the right axillary lymphadenopathy.
123	3	Vitamin D Deficiency	9	Grade 2 (Moderate)	Not related	Not applicable	Yes	Yes	Yes	Recovered/resolved	Vitamin D deficiency	Subject being replaced with Vitamin D 1000iu once daily,
123	4	Left Elbow Joint Pain	7	Grade 1 (Mild)	Not related	Not applicable	No		No	Recovered/resolved	Arthralgia	Following vaccine 3, ppt experienced left elbow joint pain on the day of vaccine administration. The pain resolved by the next day.
123	5	Skin Sensitivity	7	Grade 1 (Mild)	Related	No Change	No		No	Recovered/resolved	Sensitive skin	Skin tenderness was most problematic on subject's back with shirts by day and his sheets at night. There was no visible marks or rashes on skin. Participant reports that skin did not itch at the time and this was never medically treated. There is no history of similar post-vaccine symptoms. However, this event did not prevent participant from receiving final vaccine of the study.

											Skin tenderness resolved on day 7 post-vaccination.
123	6	Skin Sensitivity	Interim Visit	Grade 1 (Mild)	Related	No Change	No	No	Recovered/resolved	Sensitive skin	This participant describes skin sensitivity as mild to moderate in severity. It was most noticeable his back with shirts by day and his sheets at night. There are no visible no rashes and skin did not itch. This was never treated as patient reported onset dates being day 2 post-vaccination and condition is resolved by day 7. There is no past history of anything similar for this participant post other vaccines.
123	7	Skin Sensitivity	Interim Visit	Grade 1 (Mild)	Related	No Change	No	No	Recovered/resolved	Sensitive skin	This participant describes skin sensitivity as mild to moderate in severity. It was most noticeable his back with shirts by day and his sheets at night. There are no visible no rashes and skin did not itch. This was never treated as patient reported onset dates being day 2 post-vaccination and condition is resolved by day 7. There is no past history of anything similar for this participant post other vaccines.
123	8	Skin Sensitivity	Interim Visit	Grade 1 (Mild)	Related	No Change	No	No	Recovered/resolved	Sensitive skin	This participant describes skin sensitivity as mild to moderate in severity. It was most noticeable his back with shirts by

												day and his sheets at night. There are no visible no rashes and skin did not itch. This was never treated as patient reported onset dates being day 2 post-vaccination and condition is resolved by day 7. There is no past history of anything similar for this participant post other vaccines.
123	10	Skin Sensitivity	Interim Visit	Grade 1 (Mild)	Related	No Change	No		No	Recovered/resolved	Sensitive skin	Participant reported skin sensitivity. Participant came in clinic for an interim visit where a physical exam was performed only to assess his back and the reported skin sensitivity. The skin sensitivity is strictly on back and there is no rash. Participant reports this as mild.
475	1	Viral Syndrome	7	Grade 1 (Mild)	Not related	No Change	Yes	Yes	No	Recovered/resolved	Viral infection	Participant had sore throat. Rapid COVID test was negative, COVID test 3 days later was negative. Also developed a runny nose and nasal congestion.
475	2	Canker Sore	Interim Visit	Grade 2 (Moderate)	Not related	Not applicable	Yes	Yes	Yes	Recovered/resolved	Aphthous ulcer	
142	1	Panic attack	2	Grade 2 (Moderate)	Not related	Permanently discontinued	Yes	Yes	No	Recovered/resolved	Panic attack	
283	1	elevated ALT	5	Grade 1 (Mild)	Not related	No Change	No		No	Recovered/resolved	Alanine aminotransferase increased	incidental finding on scheduled labs drawn at visit 5. asymptomatic. will repeat at visit 6. Participant also ran a marathon and ran 8 more miles 4 days later. He also had 2 alcoholic beverages that day. repeat ALT closed AE.

283	2	sprain left ankle	8	Grade 2 (Moderate)	Not related	No Change	Yes	Yes	Recovered/resolved	Ligament sprain	Subject sprained left ankle while running. Subject saw a clinical provider and has a walking boot.
283	3	Presyncope	Interim Visit	Grade 1 (Mild)	Not related	Not applicable	No	No	Recovered/resolved	Presyncope	participant experienced lightheadedness/feeling of fainting but did not lose consciousness. ppt reported event at visit but experienced event outside of visit.
440	1	viral syndrome	Interim Visit	Grade 1 (Mild)	Not related	No Change	No	Yes	Recovered/resolved	Viral infection	after several days of being asymptomatic (reacto resolved), new onset of flushing, symptoms of feverishness, headache, diarrhea, no upper respiratory symptoms, not high risk exposers, high compliance with oral PREP. Had negative HIV test, seen by PCMB, ECG done and was scheduled for stress test. Participant reported he was given initial diagnosis of costochondritis. separate AE created for costochondritis.
440	2	Costochondritis	4	Grade 1 (Mild)	Not related	No Change	Yes	Yes	Recovered/resolved	Costochondritis	mild chest pressure occurred onset same as viral syndrome symptoms. Participant had chest X-ray and EKG and stress test, which were all normal. He was given diagnosis of costochondritis. chest discomfort markedly decreased, intermittent. Does not affect activities. If symptom still present in the next few months participant told to

												follow up with PCMD. ongoing and unchanged. subject confirms still ongoing and unchanged.
440	3	Asymptomatic Genitourinary Chlamydia	10	Grade 2 (Moderate)	Not related	Not applicable	Yes	Yes	Yes	Recovered/resolved	Genitourinary chlamydia infection	Asymptomatic. Tested because of possible exposure. Test positive. Treated with Doxycycline. No report of further outside testing.
440	4	Asymptomatic Rectal Chlamydia	10	Grade 2 (Moderate)	Not related	Not applicable	Yes	Yes	Yes	Recovered/resolved	Anal chlamydia infection	Asymptomatic. Tested because of possible exposure. Test Positive. Treated with Doxycycline.

Supplemental Figure 1. CONSORT diagram.

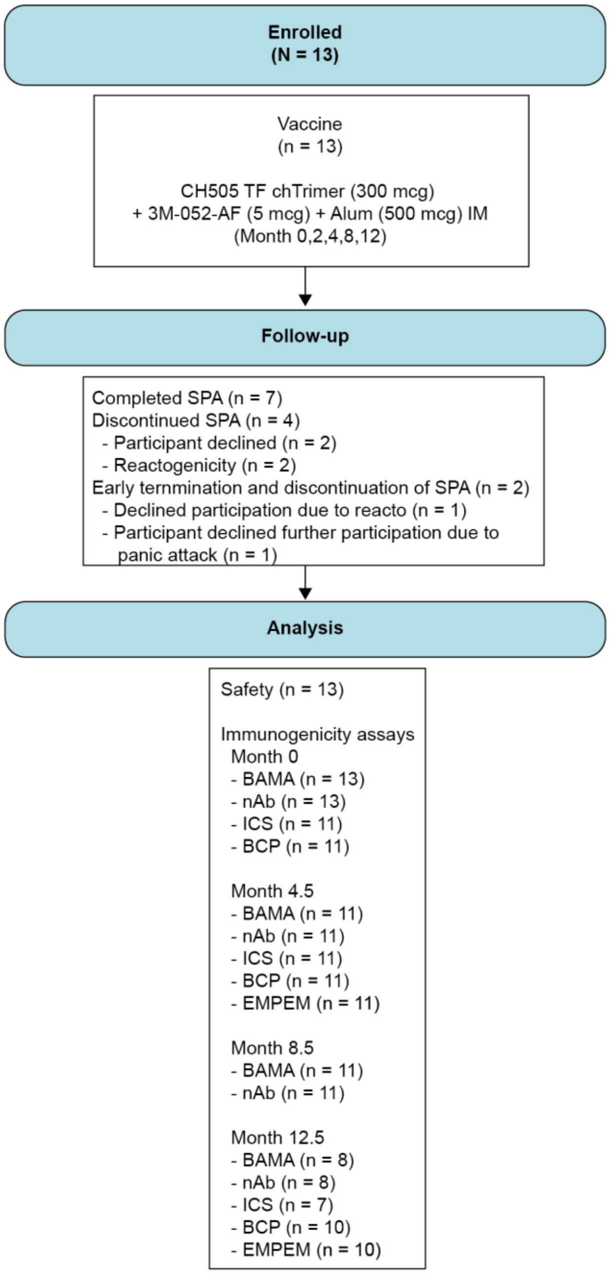

Supplemental Figure 2. Details of vaccine completion. All 13 participants are listed in rows and dose number in columns.

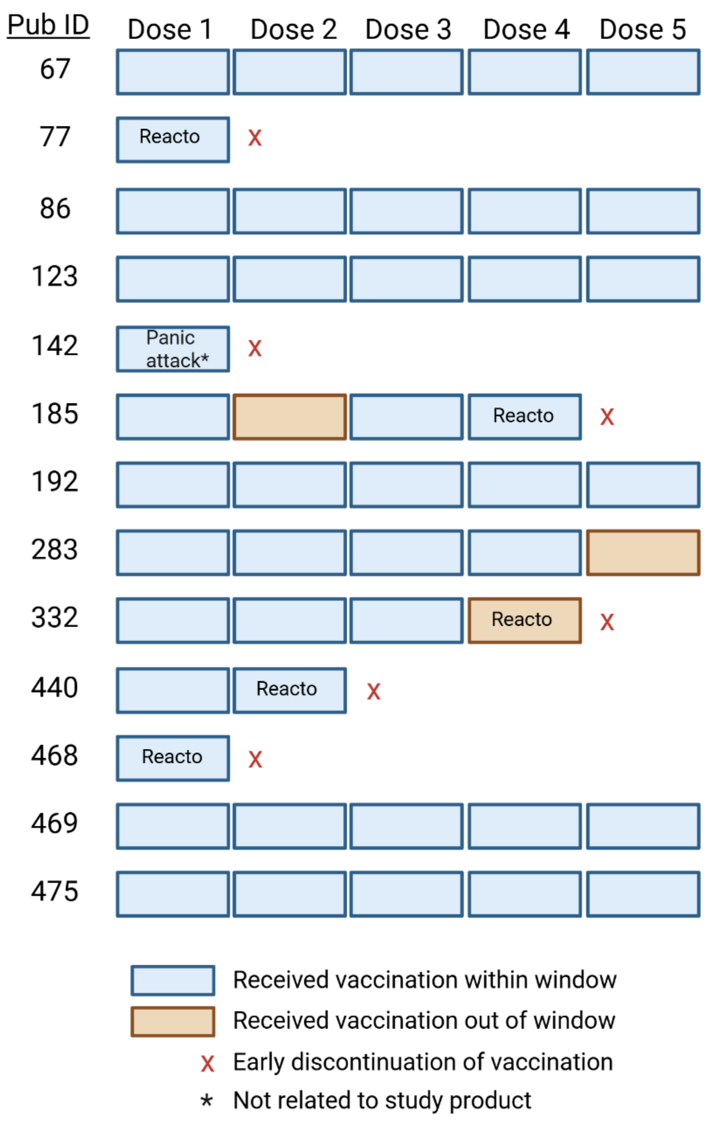

Supplemental Figure 3. CD8 T-cell and IgA responses. CD8+ T cells expressing IFN-g and/or IL-2 (A) or TNF-a (B). C) Percentage CH505+ IgA cells. Boxplots were constructed using data from all participants, with positive responders indicated in blue and non-responders in gray. Data points for each participant are connected by a gray line. The mid-line of the box denotes the median and the ends of the box denote the 25th and 75th percentiles. The whiskers that extend from the top and bottom of the box extend to the most extreme data points that are no more than 1.5 times the interquartile range (i.e., height of the box) or if no value meets this criterion, to the data extremes.

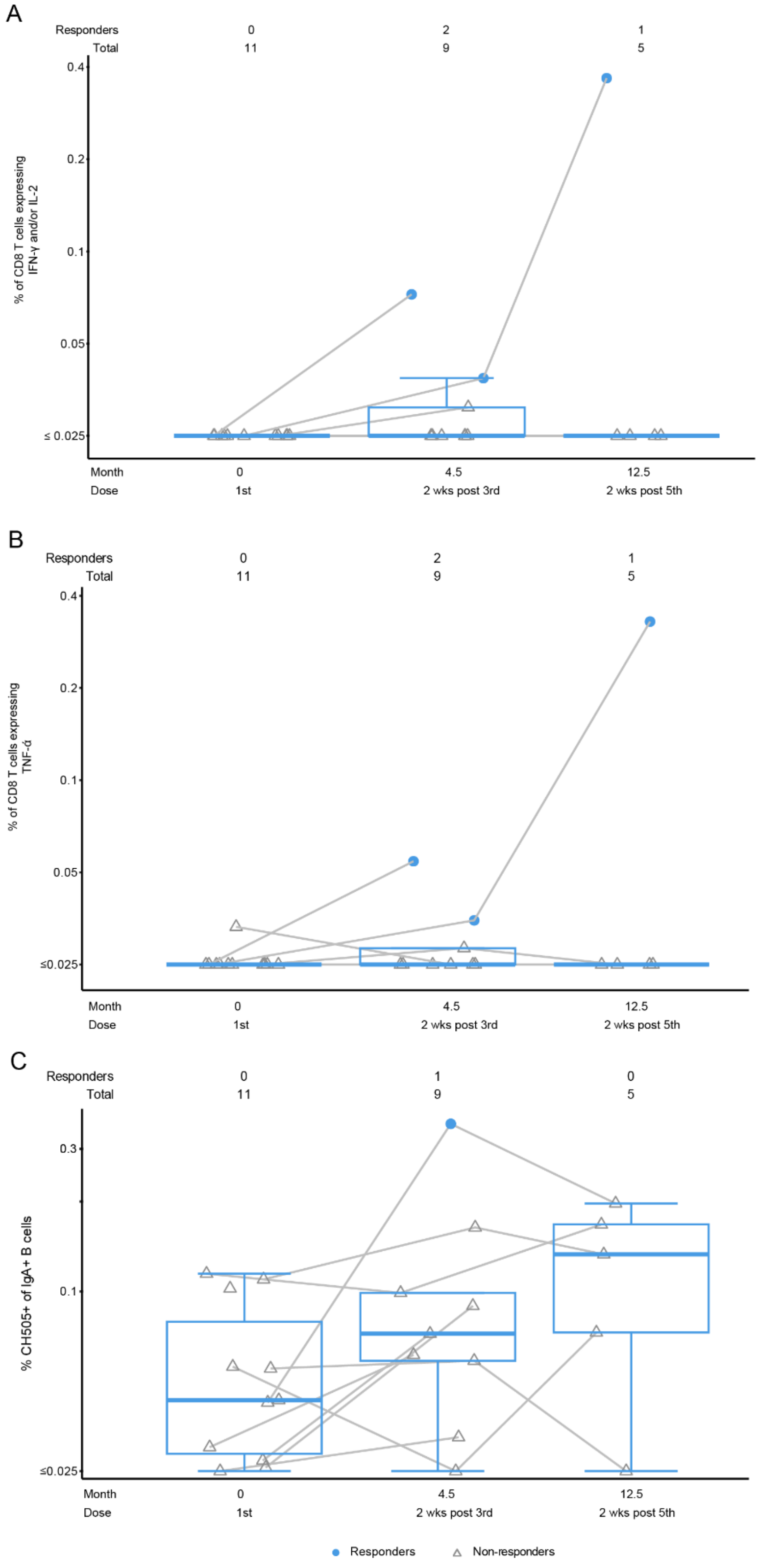

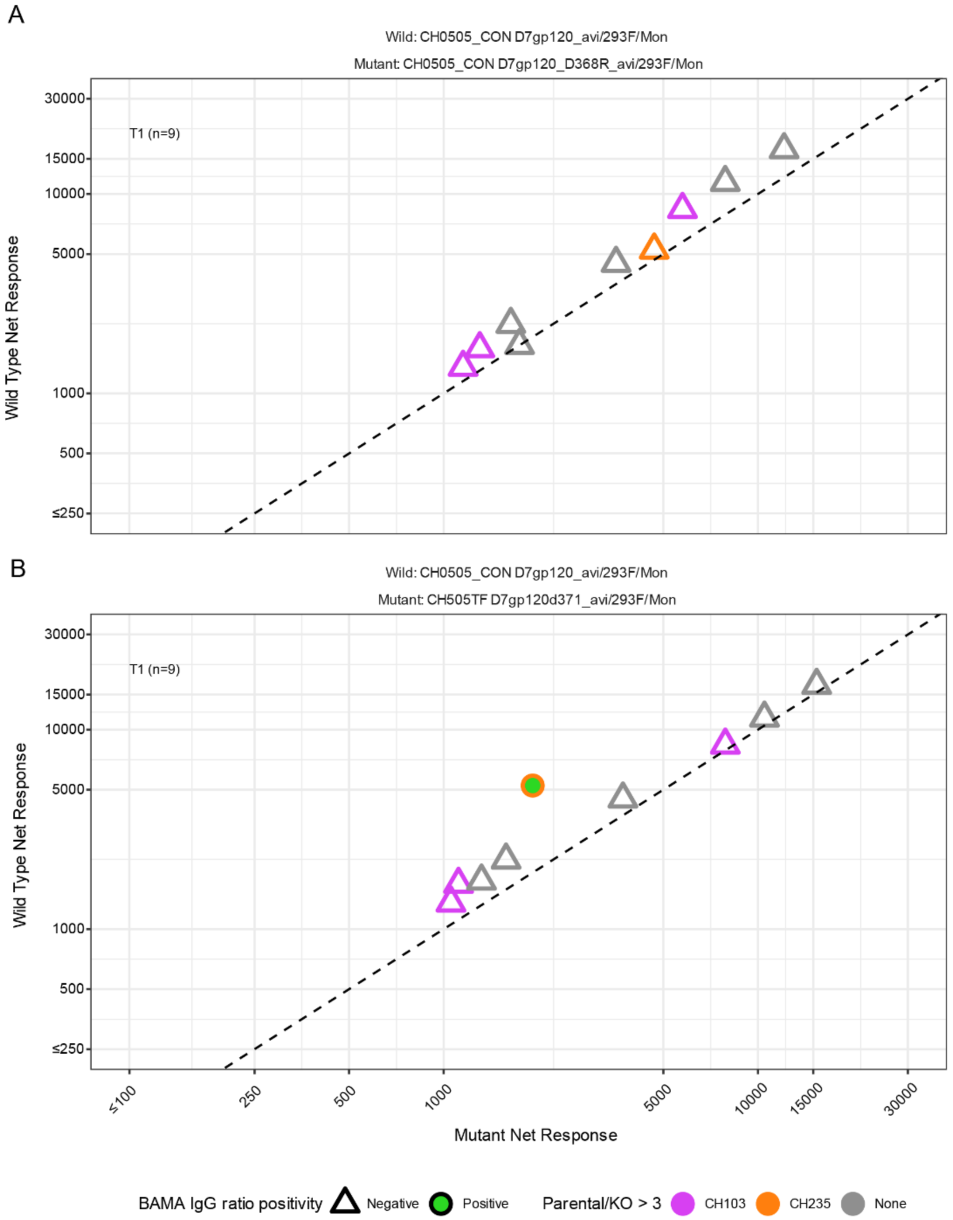

Supplemental Figure 4. Vaccine-specific binding antibodies elicited by vaccination. Specific responses were measured by BAMA at month 4.5. Wildtype CH505 gp120 was tested against sera from 9 participants against mutant gp120. A. The top panel demonstrates differential binding with a mutation intended to demonstrate CH103 like binding (D368). B. The bottom represents differential binding with a mutation intended to demonstrate CH235-like binding (D371). The purple triangles represent participants with CH103-like neutralization in polyclonal serum (e.g. a three-fold reduction in activity with CH505TF.gly4/293S (grown in GnT1- cells) compared to the corresponding strain with an additional mutation (S365P). The orange represents participants with CH235-like neutralization in polyclonal serum. The orange closed circle had a positive response by binding and neutralization assays.

Supplemental Figure 5. Differential binding of wildtype to mutant for Per Protocol participants. Seven participants are show, wildtype compared with three different mutant gp120s (G167R, N280D, and STG). Numbers above each group of graphs is the participant ID.

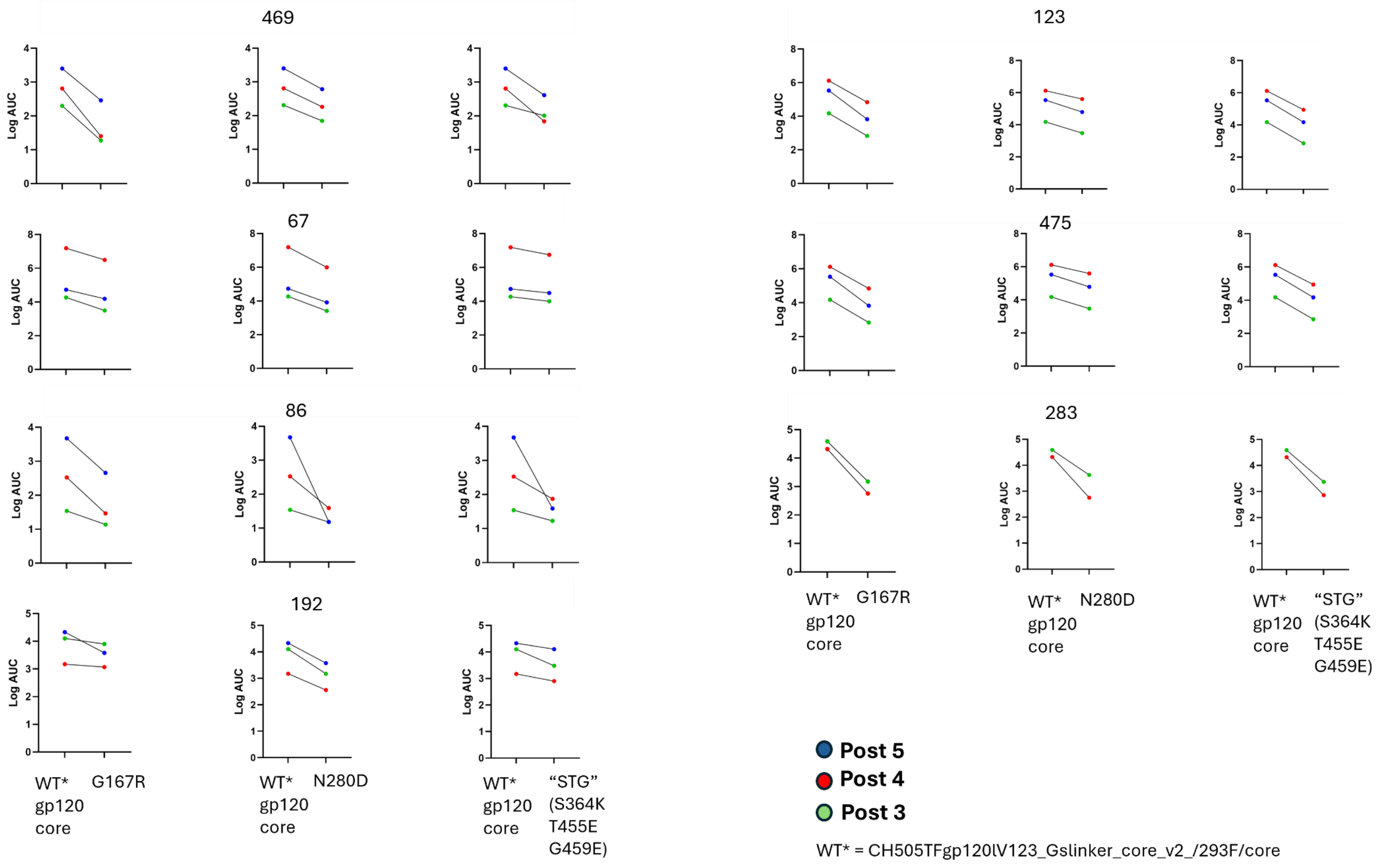

Supplement: Supplement 1 [file media-1.pdf]
